# Liver Transplantation for Pediatric Hepatocellular Carcinoma: A Systematic Review †

**DOI:** 10.3390/cancers14051294

**Published:** 2022-03-02

**Authors:** Christos D. Kakos, Ioannis A. Ziogas, Charikleia D. Demiri, Stepan M. Esagian, Konstantinos P. Economopoulos, Dimitrios Moris, Georgios Tsoulfas, Sophoclis P. Alexopoulos

**Affiliations:** 1 Surgery Working Group, Society of Junior Doctors, 15123 Athens, Greece; christos.kakos11@gmail.com (C.D.K.); ioannis.a.ziogas@vumc.org (I.A.Z.); harademiri@hotmail.com (C.D.D.); stepesag@gmail.com (S.M.E.); economopoulos@gmail.com (K.P.E.); 2 Department of Surgery, Division of Hepatobiliary Surgery and Liver Transplantation, Vanderbilt University Medical Center, Nashville, TN 37232, USA; 3 2nd Department of Pediatric Surgery, “Papageorgiou” General Hospital, Aristotle University School of Medicine, 54124 Thessaloniki, Greece; 4 Department of Surgery, Duke University Medical Center, Durham, NC 27710, USA; dimitrios.moris@duke.edu; 5 Department of Surgery, Aristotle University School of Medicine, 54124 Thessaloniki, Greece; tsoulfasg@gmail.com

**Keywords:** hepatocellular carcinoma, hepatoma, HCC, liver transplantation, pediatric, Milan criteria, living donor, fibrolamellar

## Abstract

**Simple Summary:**

Hepatocellular carcinoma is a type of primary liver cancer and the second most common type of liver cancer in children. Although partial hepatectomy can be curative, many children present with tumors that are not amenable to resection and thus the only potentially curative option is liver transplantation. In this systematic review, we have pooled the data from the worldwide literature and showed that survival after liver transplantation for pediatric hepatocellular carcinoma is favorable and many children do well even if their tumors exceed certain potentially restrictive criteria originally developed to select adults with hepatocellular carcinoma for liver transplantation.

**Abstract:**

Liver transplantation (LT) is the only potentially curative option for children with unresectable hepatocellular carcinoma (HCC). We performed a systematic review of the MEDLINE, Scopus, Cochrane Library, and Web of Science databases (end-of-search date: 31 July 2020). Our outcomes were overall survival (OS) and disease-free survival (DFS). We evaluated the effect of clinically relevant variables on outcomes using the Kaplan–Meier method and log-rank test. Sixty-seven studies reporting on 245 children undergoing LT for HCC were included. DFS data were available for 150 patients and the 1-, 3-, and 5-year DFS rates were 92.3%, 89.1%, and 84.5%, respectively. Sixty of the two hundred and thirty-eight patients (25.2%) died over a mean follow up of 46.8 ± 47.4 months. OS data were available for 222 patients and the 1-, 3-, and 5-year OS rates were 87.9%, 78.8%, and 74.3%, respectively. Although no difference was observed between children transplanted within vs. beyond Milan criteria (*p* = 0.15), superior OS was observed in children transplanted within vs. beyond UCSF criteria (*p* = 0.02). LT can yield favorable outcomes for pediatric HCC beyond Milan but not beyond UCSF criteria. Further research is required to determine appropriate LT selection criteria for pediatric HCC.

## 1. Introduction

Primary liver tumors constitute 1–2% of pediatric malignancies [1] and are the indication for 5% of all pediatric liver transplantations (LTs) (based on Organ Procurement and Transplantation Network data as of May 22, 2021). Hepatoblastoma is the most common primary liver tumor in the pediatric population (48%), followed by hepatocellular carcinoma (HCC) (27%), vascular tumors, and sarcomas [2]. Similar to hepatoblastoma, complete surgical resection is the cornerstone of treatment for pediatric HCC, especially given its chemo-resistant nature [3,4]. Compared to adult HCC, which usually arises on a cirrhotic background [5], only a small proportion of pediatric HCCs is considered to develop in a background of underlying liver disease and cirrhosis in Western countries [5,6,7]. Furthermore, pediatric HCC often presents at an advanced stage and with a larger tumor size compared to adult HCC patients, frequently exceeding both Milan and University of California San Francisco (UCSF) criteria [1], which were originally developed to determine transplantability for adult HCC [8]. LT performed for oncologic purposes for pediatric HCC can not only remove the diseased liver background predisposing to HCC in case of underlying cirrhosis or metabolic disorder, but also decrease the risk of liver failure after liver resection for large HCCs.

Studies using data from the National Cancer Database (NCDB) and the Surveillance, Epidemiology, and End Results (SEER) database have shown that LT is associated with better outcomes compared to partial hepatectomy for pediatric HCC [9,10]. Small case series from reference centers have shown that LT can lead to favorable long-term outcomes for pediatric HCC even beyond of the Milan criteria [11,12]. However, no study has summarized the worldwide experience of LT for HCC in the pediatric population.

In this systematic literature review, we aimed to summarize all available data on the clinicopathological characteristics and oncological outcomes after LT for pediatric HCC.

## 2. Materials and Methods

### 2.1. Study Design, Search Strategy and Eligibility Criteria

The present systematic review of the literature was performed based on the Preferred Reporting Items for Systematic Reviews and Meta-Analysis (PRISMA) statement (Table S1) [13]. Patient consent and Institutional Review Board approval were not required because this was a systematic review of already published articles. This study is registered with the Research Registry (www.researchregistry.com, accessed on 27 February 2022), and its unique identifying number is: reviewregistry1310.

The Population/Participants, Intervention, Comparison, Outcome, and Study Design (PICOS) framework was used to define the inclusion criteria:

- Participants: Patients < 18 years of age of any sex or race undergoing LT for HCC
- Interventions: LT
- Comparison: Not applicable
- Outcomes: Overall Survival (OS) and disease-free survival (DFS)
- Study Design: randomized clinical trials or non-randomized (either prospective or retrospective) clinical studies, case series, or case reports.

Excluded studies met at least one of the following criteria: (i) articles published in a language other than English, (ii) studies irrelevant to LT for HCC in children, (iii) studies limited to adult patients (≥18 years old), (iv) articles including both adult and pediatric patients and not providing data separately for those < 18 years old, (v) studies which did not specify if pediatric patients were included, (vi) studies with patients who underwent multivisceral transplantations or retransplantations, (vii) in vitro studies, (viii) animal studies, (ix) narrative or systematic reviews and meta-analyses, (x) letters to the editor, comments, errata, book chapters without primary patient data, and (xi) published abstracts without a full text. In the case of patient overlap, the most recent study or the one with the largest population was included. However, when variables of interest were presented in more than one eligible study, data extraction was performed from all without patient populations being summed, as they constituted additional data on the same populations.

Eligible studies were identified through a comprehensive search of the MEDLINE (through PubMed), Scopus, Cochrane Library and Web of Science databases (end-of-search date: 31 July 2020) by two independent researchers (C.D.K., C.D.D.) using the Covidence reference and article manager software [14]. We used the following algorithm: (liver transplant*) AND (hcc OR hepatocellular carcinoma OR hepatoma) AND (child* OR pediatr*). No publication date or any other search filters were applied. Any disagreements were identified and resolved through quality control discussions with the other two authors (S.M.E., I.A.Z.). We also hand-searched the reference lists of the included articles and other published systematic reviews for potentially relevant, missed studies according to the “snowball” methodology [15].

### 2.2. Data Tabulation and Extraction

Data tabulation and extraction was performed using a standardized, pre-piloted form by two reviewers (C.D.K., C.D.D.) independently, and any disagreements were discussed with two other reviewers (S.M.E., I.A.Z.). The following variables of interest were extracted from the included articles: study data (author, publication year, study design, location, study period, number of patients), patient data (age at the time of LT, sex, pre-LT diagnosis or incidental HCC, underlying liver disease, cirrhosis), graft type, prior resection or transarterial chemoembolization (TACE), neoadjuvant or adjuvant chemotherapy, a-fetoprotein (AFP) level in μg/mL, histological type of HCC (fibrolamellar vs. non-fibrolamellar), tumor size in cm, number of nodes, presence of metastasis, presence of macro- or microvascular invasion, whether the patient was within or beyond the Milan criteria [8], the UCSF criteria [16], the alpha-fetoprotein-adjusted-to-HCC-size (AFP-UTS) criteria [17], postoperative complications (graft rejection, infection, bleeding, hepatic artery thrombosis or other) and survival outcomes (OS, DFS, cause of death) after LT.

The published Kaplan–Meier curves or individual patient data tables from the included articles were used for survival data extraction. We downloaded and digitized the Kaplan–Meier curve images from the included studies to extract the survival step function values and timings of the steps and individual patient survival information was obtained based on the numerical solutions to the inverted Kaplan–Meier product-limit equations. When not available, the censoring pattern was assumed to be non-informative and constant within each time interval, but when number-at-risk tables or total number of events were available, they were used to improve data accuracy.

### 2.3. Statistical Analysis

Continuous data were reported in means and standard deviation (SDs), while categorical data were reported in frequencies and percentages. When continuous data were provided in median and range, the method by Hozo et al. [18] to calculate the mean and SD was used, and when continuous data were provided in median and interquartile range, the method by Wan et al. [19] was used instead. Since not all studies reported on all variables of interest, relative rates were calculated according to the available data and based on the Cochrane Handbook principles [20]. OS and DFS were defined as the time interval from the LT date to the date of patient death or recurrence, respectively, or last patient contact. The 1-, 3-, and 5-year OS and DFS rates were calculated using the Kaplan–Meier method. We further examined the effect of sex, pre-LT vs. incidental HCC diagnosis, cirrhosis, graft type, neoadjuvant and adjuvant chemotherapy, non-fibrolamellar vs. fibrolamellar histology, macro- and microvascular invasion, and the Milan, UCSF, and AFP-UTS criteria on OS using the log-rank test. Statistical analyses were conducted with the computing environment R version 3.6.3 [21], all *p*-values were two-sided, and a *p* < 0.05 was considered to be statistically significant.

## 3. Results

### 3.1. Study Selection and Characteristics

**Figure 1:**
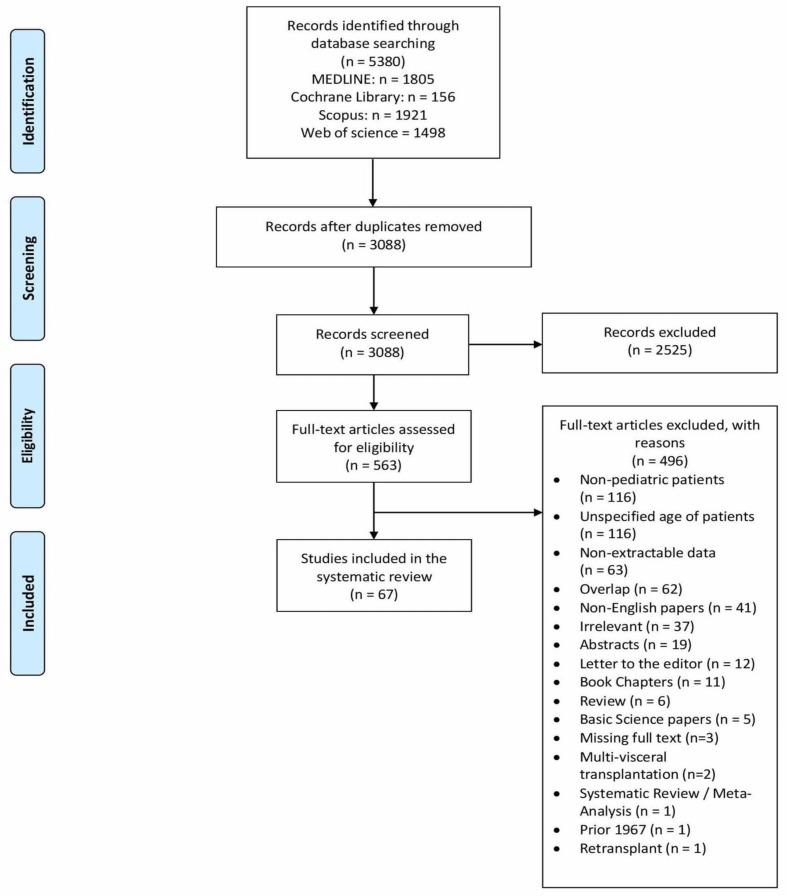
PRISMA flow diagram.

**Figure 2:**
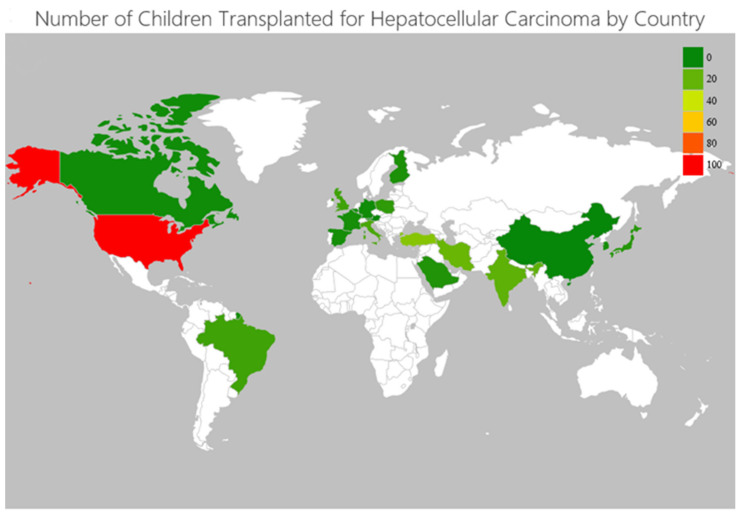
Geographical map representation of children transplanted for hepatocellular carcinoma worldwide.

Our initial search yielded 5380 potentially relevant records. After screening titles and abstracts, 563 articles were retrieved for full-text evaluation. Ultimately, 67 non-overlapping studies [11,12,22,23,24,25,26,27,28,29,30,31,32,33,34,35,36,37,38,39,40,41,42,43,44,45,46,47,48,49,50,51,52,53,54,55,56,57,58,59,60,61,62,63,64,65,66,67,68,69,70,71,72,73,74,75,76,77,78,79,80,81,82,83,84,85,86] reporting on 245 patients were included in our systematic review (Table 1 and Figure 1). The distribution of patients by country is shown in Figure 2. The mean age of pediatric patients at the time of LT was 8.2 ± 5.3 years. The diagnosis of HCC was established pre-LT in 61.2% (*n* = 115/188) and incidentally based on the pathology of the liver explant in 38.8% (*n* = 73/188). The data on the primary LT indication in patients with incidental HCC were available for 63 of the 73 patients, and the most common were tyrosinemia (41.3%, *n* = 26/63), and biliary atresia (14.3%, *n* = 9/63). Overall, underlying liver disease was present in 80.9% (*n* = 183/226) of patients, with the most common being tyrosinemia (34.1%, *n* = 77/226) and biliary atresia (11.1%, *n* = 25/226). The majority of patients had cirrhosis (79.6%, *n* = 129/162). Most patients underwent deceased donor liver LT (59.1%, *n* = 81/137), with 60 receiving whole and 21 split grafts, while 56 patients (40.9%, *n* = 56/137) underwent living donor LT.

Prior resection before LT was performed in 9.2% (*n* = 15/163) and the indications for LT in this setting were recurrence after liver resection (*n* = 9/15), incomplete/margin-positive liver resection (*n* = 2/15), or not specified (*n* = 4/15). Prior TACE was performed in 10.8% (*n* = 16/148) and pre-LT chemotherapy was administered in 31.6% (*n* = 50/158) with the most common agents being cisplatin and doxorubicin. Adjuvant chemotherapy was administered in 23.8% (*n* = 30/126) of patients, with cisplatin and doxorubicin again being the most preferred choices. The mean AFP level was 37,774.2 μg/mL. Fibrolamellar histology was seen in 12.9% (*n* = 15/116), while 58.1% (*n* = 93/160) of patients had HCC beyond the Milan criteria and 47.3% (*n* = 70/148) beyond the UCSF criteria. Patient characteristics are shown in Table 2.

### 3.2. Synthesis of Results

#### 3.2.1. Complications

Post-LT complications were encountered in 60.9% (*n* = 39/64), the most common of which were infection in 23.1% (*n* = 12/52), rejection in 19.5% (*n* = 16/82), and hepatic artery thrombosis in 7.0% (*n* = 4/57). Retransplantation was reported in 5.6% (*n* = 6/108) of all cases.

#### 3.2.2. Disease-Free Survival

**Figure 3:**
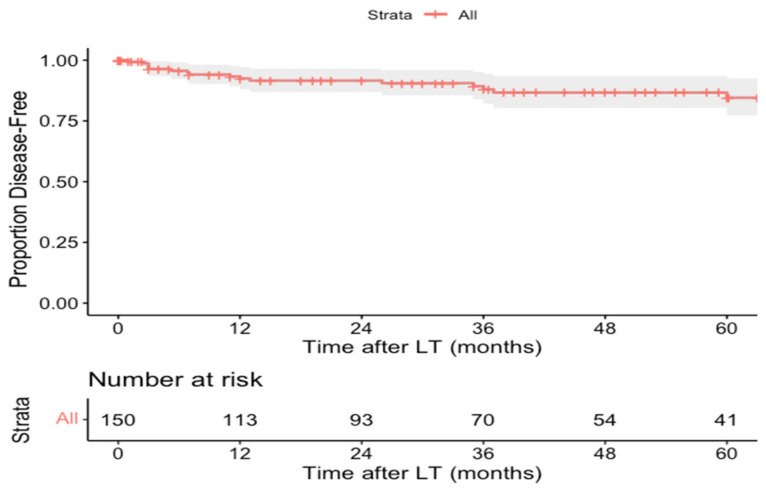
Kaplan–Meier disease-free survival curve of pediatric hepatocellular carcinoma liver transplant recipients.

Tumor recurrence was reported in 16.2% (*n* = 35/216) over a mean follow-up of 38.6 ± 34.7 months. Data regarding the site of recurrence were available in 19 of the 35 patients experiencing recurrence and these included the lungs (63.2%, *n* = 12/19; one patient also had recurrence to the paraaortic lymph nodes and one patient also had recurrence to the abdomen, not otherwise specified), liver (26.3%, *n* = 5/19; one patient also had recurrence to the diaphragm and retroperitoneum and another patient to the stomach and pelvis), paraaortic lymph nodes (5.3%, *n* = 1/19), and pelvis (5.3%, *n* = 1/19). DFS data were available for 150 patients and the 1-, 3-, and 5-year DFS rates were 92.3% (95% CI: 88.4–96.8%), 89.1% (95% CI: 84.3–93.9%), and 84.5% (95% CI: 80.3–91.8%), respectively (Figure 3). The limited availability of data did not allow us to examine the effect of certain variables of interest on DFS.

#### 3.2.3. Overall Survival

**Figure 4:**
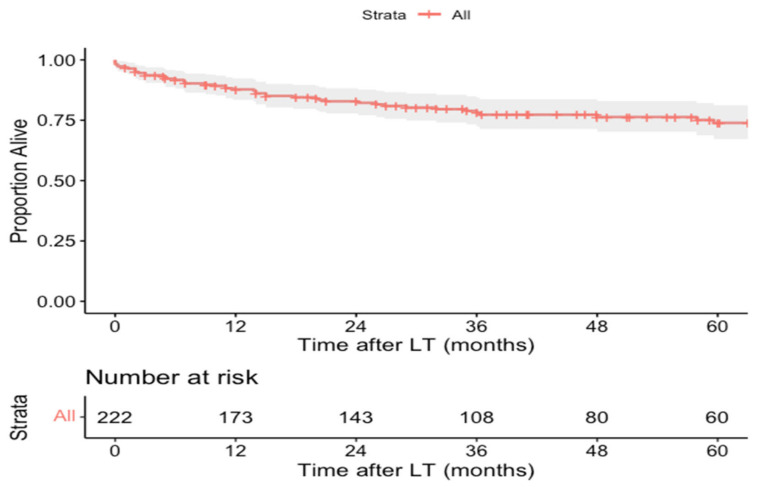
Kaplan–Meier overall survival curve of pediatric hepatocellular carcinoma liver transplant recipients.

Sixty of the two hundred and thirty-eight patients (25.2%) died over a mean follow-up of 46.8 ± 47.4 months. The most common cause of death was tumor recurrence (36.7%, *n* = 22/60), followed by chronic allograft rejection (8.3%, *n* = 5/60), sepsis (8.3%, *n* = 5/60; one also had gastrointestinal bleeding), primary non-function (5.0%, *n* = 3/60), and cytomegalovirus infection (3.3%, *n* = 2/60) (Table 3). OS data were available for 222 patients and the 1-, 3-, and 5-year OS rates were 87.9% (95% CI: 83.9–92.4%), 78.8% (95% CI: 73.4–85.0%), and 74.3% (95% CI: 67.5–81.1%), respectively (Figure 4).

#### 3.2.4. Additional Analyses

**Figure 5:**
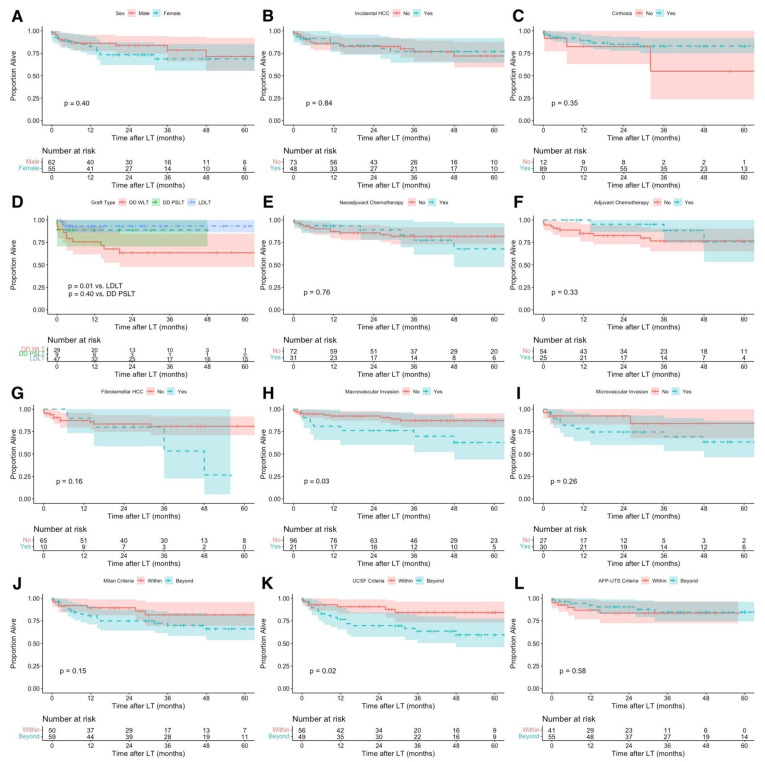
Kaplan–Meier overall survival curve of pediatric hepatocellular carcinoma liver transplant recipients: (**A**) males vs. females, (**B**) non-incidental (pre-LT diagnosis) vs. incidental HCC, (**C**) no cirrhosis vs. cirrhosis, (**D**) deceased donor whole liver transplant (DD WLT) vs. deceased donor partial/split liver transplant (DD PSLT) vs. living donor liver transplant (LDLT), (**E**) no neoadjuvant chemotherapy vs. neoadjuvant chemotherapy, (**F**) no adjuvant chemotherapy vs. adjuvant chemotherapy, (**G**) non-fibrolamellar vs. fibrolamellar HCC histological type, (**H**) no macrovascular invasion vs. macrovascular invasion, (**I**) no microvascular invasion vs. microvascular invasion, (**J**) within vs. beyond the Milan criteria, (**K**) within vs. beyond the University of California San Francisco (UCSF) criteria, (**L**) within vs. beyond the alpha-fetoprotein-adjusted-to-HCC-size (AFP-UTS) criteria.

No statistically significant differences were observed in OS when patients were stratified by sex (*p* = 0.40), incidental HCC diagnosis (*p* = 0.84), and cirrhosis (*p* = 0.35) (Figure 5A–C). Notably, inferior OS was observed in children who received deceased donor whole graft compared with children who received living donor graft (*p* = 0.01), while no difference was observed between children who received deceased donor whole vs. partial/split graft (*p* = 0.40) (Figure 5D). No statistically significant differences were also observed in OS when patients were stratified by receipt of neoadjuvant (*p* = 0.76) or adjuvant chemotherapy (*p* = 0.33) and HCC histological type (non-fibrolamellar vs. fibrolamellar) (*p* = 0.16) (Figure 5E–G). Children with HCC with macrovascular invasion had inferior OS compared with children without macrovascular invasion (*p* = 0.03), while no difference in OS was observed regarding microvascular invasion (*p* = 0.26) (Figure 5H,I). Although no difference was observed between children transplanted within vs. beyond the Milan criteria (*p* = 0.15), superior OS was observed in children transplanted within vs. beyond the UCSF criteria (*p* = 0.02) (Figure 5J,K). No statistically significant difference was observed between children transplanted within vs. beyond the AFP-UTS criteria (*p* = 0.58) (Figure 5L).

## 4. Discussion

HCC represents an aggressive tumor with dismal prognosis without surgical resection. There are age-dependent differences in epidemiology, histology, and response to treatment. The biological behavior of liver tumors in pediatric patients even for similar histological groups is different, so surgeons should follow a distinct strategy. Current evidence suggest that complete excision of the tumor is essential to achieve long-term survival [87]. This can be accomplished either by partial liver resection or LT. Considering the fact that pediatric HCC not uncommonly presents at an unresectable stage, LT constitutes the only potential therapeutic option for these patients. Several selection criteria have been proposed to identify the most appropriate candidates who will benefit from LT for HCC. The most widely adopted are the Milan criteria established initially for adult cirrhotic HCC patients (one tumor ≤ 5 cm or up to 3 tumors each ≤ 3 cm, with no vascular invasion or metastatic disease) [8]. However, several attempts have been made to expand these criteria and offer LT to more potentially eligible candidates (i.e., UCSF criteria, Toronto criteria, AFP-UTS, etc.) [88]. The UCSF criteria are defined as one tumor ≤ 6.5 cm or up to three tumors with the largest tumor diameter ≤ 4.5 cm and total tumor diameter < 8 cm, with no vascular invasion or metastatic disease [16]. Additionally, based on the “Metroticket 2.0” calculation model, the AFP-UTS criteria (“Up To Seven” for AFP < 200 ng/mL, “Up To Five” for AFP 200–400 ng/mL, and “Up To Four” for AFP 400–1000 ng/mL) were proposed for patient selection [17]. 

Herein, we present the largest cohort of pediatric LT recipients for HCC, reporting on demographic characteristics, clinicopathological features and prognosis of HCC in patients under 18 years old. More than 80% of the children undergoing LT included in our systematic review had underlying liver disease and nearly 80% had a cirrhotic liver. Although pediatric HCC is less likely to arise in the context of underlying liver disease compared to adult HCC, our finding may be attributed to selection bias in that children with HCC and underlying liver disease may be more likely to undergo LT rather than liver resection; it may also be due to publication bias, in that clinicians may be more likely to publish a report with a child undergoing LT for HCC on a background of a rare liver condition vs. for a de novo HCC. Our analysis showed that the 5-year OS rate in children undergoing LT was 74%, which is compatible with several series reporting 5-year survival of 58–88% [9,89,90] and equivalent to that of hepatoblastoma [90]. We tried to elucidate the effect of several risk factors on OS. According to our results, there was no difference in OS between children with versus without cirrhosis. Additionally, patients with fibrolamellar histology often exhibit superior survival compared to conventional HCC histology [91], yet our results did not confirm this notion, mostly because of the small sample size. Moreover, we found no statistically significant difference between children transplanted within vs. beyond the Milan criteria, in accordance with distinct single-center reports [11,12] and US registry data [9]. However, a statistically significant difference was observed between children transplanted within vs. beyond the UCSF criteria. The Milan criteria appear to be too restrictive for pediatric patients, and thus further assessment of the UCSF criteria in a prospective study may improve patient selection. Notably, no difference in OS was observed when comparing children within vs. beyond the AFP-UTS criteria, and further evaluation of the role of AFP in the pediatric population is also warranted. However, future research should focus on the role of molecular biomarkers, such as circulating tumor DNA [92], to better identify children with HCC beyond current restrictive criteria who would benefit from LT. Even though certain patients with metastatic HCC have been transplanted and have achieved long-term survival [10], performing LT for metastatic pediatric HCC is not recommended.

Living donor grafts showed a statistically significant survival benefit vs. deceased donor whole grafts. A retrospective cohort study from Kyushu University, Japan showed comparable tumor recurrence rates but improved OS in favor of grafts from living donors when compared with grafts from deceased donors [93]. The authors attributed the difference in OS to nontumor-related factors associated with more stringent eligibility criteria for living donation and donor-related factors [93]. Moreover, the availability of living donors allows surgeons to perform LT with more liberal criteria for tumor staging, and thus living related grafts could be preferred whenever available. Additionally, a European Liver Transplant Registry report (ELTR) showed that children with HCC in the setting of inherited liver disease had superior survival compared to children with HCC in the setting on uninherited liver disease [89]. This finding may be explained due to the fact that patients with inherited liver disease are generally followed up at more regular intervals and may thus have better chances of earlier HCC detection and earlier LT evaluation. On the contrary, patients with de novo HCC are usually diagnosed at an advanced stage.

Similar to prior studies [9,10,11,12,22], our findings could not provide convincing evidence of a survival benefit with either neoadjuvant or adjuvant chemotherapy for pediatric HCC. These findings are also in accordance with the SIOPEL 1, 2, and 3 studies [3,4], which showed that the most significant factor for improved survival is achieving margin-negative resection. The current state of knowledge according to most experts in the field is that chemotherapy has no role for resectable tumors at diagnosis [4]. A currently ongoing, prospective clinical trial (Pediatric Hepatic Malignancy International Therapeutic Trial) investigates the use of neoadjuvant chemotherapy only in patients with unresectable or metastatic tumors and may further clarify whether chemotherapy has a role in the management of HCC in the pediatric population [94]. 

Nevertheless, there are certain limitations that should be taken into consideration. As with any systematic review, some of the articles did not report on all variables of interest, and thus all relative rates were calculated according to the availability of data. The lack of reporting on specific risk factors in several studies precluded us from estimating the effect of these parameters on OS, while the limited availability of data did not allow us to examine the effect of certain variables of interest on DFS. In addition, there is a slight likelihood that some of patients transplanted in an older era might have had undiagnosed mild variants of PFIC instead of a true de novo HCC. Lastly, our long-term follow-up is limited, mainly due to missing outcome data in some studies.

## 5. Conclusions

The majority of children undergoing LT for HCC have underlying liver disease, while OS does not seem to differ between children with and without cirrhosis. LT offers complete margin-negative resection resulting in long-term survival in children with HCC. Children beyond the Milan criteria showed equivalent results when compared with those within the Milan criteria. However, children beyond the UCSF criteria appear to have worse OS when compared with those within the UCSF criteria, while no difference in OS was observed in children transplanted within vs. beyond the AFP-UTS criteria. Although further evaluation of the role of AFP in risk stratification may be useful, the future lies in continued investigation of novel molecular markers. A living graft may yield superior survival outcomes and may thus be the preferred option when available. Finally, a multidisciplinary team involving transplant surgeons, pediatric oncologists and hepatologists should be involved in the evaluation of these pediatric patients to optimize outcomes.

## Figures and Tables

**Table 1:** Studies included in this systematic review.

Author	Transplant Center	Country	*n*
D’Souza, 2020 [22]	Cincinnati Children’s Hospital, Cincinnati	USA	11
Liu, 2020 [23]	Renji Hospital, Shanghai Jao Tong University, Shanghai	China	1
Karaca, 2019 [24]	Izmir Kent Hospital, Izmir	Turkey	6
Waich, 2019 [25]	Medical University of Innsbruck, Innsbruck	Austria	1
Valamparampil, 2019 [26]	Institute of Liver Disease and Transplantation, Chennai	India	3
Kumar, 2019 [27]	King Faisal Specialist Hospital, Riyadh	Saudi Arabia	1
Kang, 2019 [28]	Asan Liver Center, Seoul	South Korea	1
Timothy, 2019 [29]	Mayo Clinic, Rochester	USA	1
Tiusanen, 2019 [30]	Helsinki University Hospital, Helsinki	Finland	5
Cowell, 2019 [31]	Baylor College of Medicine, Houston	USA	4
Chen, 2018 [32]	Stanford University Medical Center, Palo Alto	USA	1
Vinayak, 2017 [33]	University of Pittsburgh Medical Center, Pittsburgh	USA	25
Kohorst, 2017 [34]	Mayo Clinic, Rochester	USA	2
Khan, 2017 [35]	Washington University, Saint Louis	USA	2
Geramizadeh, 2017 [36]	Shiraz University of Medical Sciences, Shiraz	Iran	20
DePasquale, 2017 [37]	Bambino Gesu Pediatric Hospital, Rome	Italy	3
Troisi, 2017 [38]	Ghent University Medical School, Ghent	Belgium	1
Haberal, 2017 [39]	Baskent University, Ankara	Turkey	11
Viswanathan, 2017 [40]	Children’s Hospital at Montefiore, New York	USA	1
Benedict, 2017 [41]	Yale University School of Medicine, New Haven	USA	1
Friend, 2017 [42]	University of California, Los Angeles	USA	2
Imseis, 2017 [43]	University of Texas Health Science Center, Houston	USA	1
Triana, 2016 [44]	Hospital Universitario La Paz, Madrid	Spain	4
Shah, 2016 [45]	Bai Jerbai Wadia Hospital, Mumbai	India	1
Palaniappan, 2016 [46]	Institute of Liver Disease and Transplantation, Chennai	India	12
Park, 2016 [47]	Seoul National University College of Medicine, Seoul	South Korea	1
Picoraro, 2016 [48]	Columbia University Medical Center, New York	USA	1
Pham, 2015 [12]	Stanford University Medical Center, Palo Alto	USA	10
Abdelfattah, 2015 [49]	King Fasai Specialist Hospital, Riyadh	Saudi Arabia	4
Yu, 2015 [50]	Seoul National University College of Medicine, Seoul	South Korea	1
Samuk, 2015 [51]	University of Miami Miller School of Medicine, Miami	USA	3
Neto, 2014 [52]	Hospital Sirio-Libares, Hospital AC Camargo, Sao Paulo	Brazil	12
Bartlett, 2014 [53]	Birmingham Children’s Hospital, Birmingham	UK	1
Malik, 2014 [54]	Children’s Hospital of Philadelphia, Philadelphia	USA	1
AlSaloom, 2013 [55]	Qassim University, Al-Qassim	Saudi Arabia	1
Bhatia, 2013 [56]	Indraprastha Apollo Hospital, New Delhi	India	2
Yeop, 2012 [57]	Birmingham Children’s Hospital, Birmingham	UK	1
Schmid, 2012 [58]	Multicenter	Germany	2
Kim, 2012 [59]	Samsung Medical Center, Seoul	South Korea	1
Hadzic, 2011 [60]	King’s College Hospital, London	UK	5
Romano, 2011 [61]	San Gerardo Hospital, Milan	Italy	10
Ismail, 2009 [11]	Children’s Memorial Health Institute, Warsaw	Poland	9
Masurel Paulet, 2008 [62]	Multicenter	France	2
Gonzalez-Peralta, 2009 [63]	University of Florida, Gainesville	USA	1
Iida, 2009 [64]	University of Florida, Gainesville	USA	1
Riva, 2008 [65]	ISMETT, Palermo	Italy	1
Nara, 2008 [66]	Hirosaki University School of Medicine, Hirosaki City	Japan	1
Brunati, 2007 [67]	Saint-Luc University Clinics, Brussels	Belgium	1
Morotti, 2007 [68]	Mount Sinai School of Medicine, New York	USA	1
Freisinger, 2006 [69]	Children’s Hospital and Institute of Medical Genetics	Germany	1
Buyukpamcku, 2006 [70]	Hacettepe Uni Faculty of Medicine, Hacettepe	Turkey	3
Scheers, 2005 [71]	Saint-Luc University Clinics, Brussels	Belgium	2
Nart, 2003 [72]	Ege University Medical School, Izmir	Turkey	6
Kawasaki, 2002 [73]	Shinshu University, Matsumoto	Japan	3
Tatekawa, 2001 [74]	Kyoto University, Kyoto	Japan	2
El-Gazzaz, 2000 [75]	Queen Elizabeth Hospital, Birmingham	UK	2
Superina, 1996 [76]	Hospital for Sick Children, Toronto	Canada	3
Ojogho, 1996 [77]	Stanford University Medical Center, Palo Alto	USA	7
Broughan, 1994 [78]	Cleveland Clinic Foundation, Cleveland	USA	2
Esquivel, 1994 [79]	California Pacific Medical Center, San Francisco	USA	5
Kawarasaki, 1994 [80]	University of Shinshu Hospital, Matsumoto	Japan	1
Yandza, 1993 [81]	Hopital Bicetre, Paris	France	2
Salt, 1992 [82]	Addenbrooke’s Hospital, Cambridge	UK	2
Ismail, 1990 [83]	Queen Elizabeth Hospital, Birmingham	UK	1
Dehner, 1989 [84]	University of Minnesota, Minneapolis	USA	1
Finlay, 1987 [85]	University of Wisconsin, Madison	USA	1
Iwatsuki, 1985 [86]	University of Colorado, Denver	USA	7

**Table 2:** Systematic Review Cohort Characteristics.

Variable	Total (*n* = 245)
**Clinical Characteristics**	
Age at liver transplant (years) (*n* = 185)	8.1 ± 5.3
Sex (*n* = 153)	
Female	74 (48.4%)
Male	79 (51.6%)
Graft type (*n* = 137)	
Deceased whole	60 (43.8%)
Deceased partial/split	21 (15.3%)
Living	56 (40.9%)
Underlying liver disease overall (*n* = 226)/in patients with incidental HCC (*n* = 63)	
Tyrosinemia	77 (34.1%)/26 (41.3%)
Biliary Atresia	25 (11.1%)/9 (14.3%)
PFIC	19 (8.4%)/5 (7.9%)
Hepatitis B Virus Infection	16 (7.0%)/1 (1.6%)
Alagille Syndrome	9 (3.9%)/4 (6.3%)
Hepatitis C Virus Infection	4 (1.8%)/1 (1.6%)
Idiopathic Neonatal Hepatitis	3 (1.3%)/1 (1.6%)
A1AT deficiency	2 (0.9%)/0 (0.0%)
Glycogen Storage Disease	2 (0.9%)/0 (0.0%)
Abernethy Syndrome	2 (0.9%)/2 (3.2%)
Meso-caval shunt	2 (0.9%)/2 (3.2%)
DGUOK deficiency	2 (0.9%)/0 (0.0%)
MPV17 deficiency	2 (0.9%)/0 (0.0%)
MRCD	2 (0.9%)/2 (3.2%)
Primary Sclerosing Cholangitis	1 (0.4%)/1 (1.6%)
Autoimmune Hepatitis	1 (0.4%)/0 (0.0%)
Giant Cell Hepatitis	1 (0.4%)/1 (1.6%)
Non-ABC Hepatitis	1 (0.4%)/1 (1.6%)
Wilson Disease	1 (0.4%)/0 (0.0%)
Hemochromatosis	1 (0.4%)/1 (1.6%)
Niemann Pick Disease	1 (0.4%)/1 (1.6%)
Caroli’s Disease	1 (0.4%)/1 (1.6%)
Fibrocystic Disease	1 (0.4%)/1 (1.6%)
Kabuki Syndrome	1 (0.4%)/0 (0.0%)
Turner Syndrome	1 (0.4%)/1 (1.6%)
IFALD	1 (0.4%)/1 (1.6%)
CESD	1 (0.4%)/0 (0.0%)
MDR3 deficiency	1 (0.4%)/1 (1.6%)
NCL	1 (0.4%)/0 (0.0%)
ADA	1 (0.4%)/0 (0.0%)
Cirrhosis (*n* = 162)	129 (79.6%)
**Tumor characteristics**	
Tumor type (*n* = 116)	
Non-Fibrolamellar	101 (87.1%)
Fibrolamellar	15 (12.9%)
Multiple nodules (*n* = 198)	114 (57.6%)
Metastasis at Diagnosis (*n* = 200)	12 (6.0%)
Microvascular Invasion (*n* = 86)	42 (48.8%)
Macrovascular Invasion (*n* = 150)	26 (17.3%)
Beyond Milan Criteria (*n* = 160)	93 (58.1%)
Beyond UCSF Criteria (*n* = 148)	70 (47.3%)
**Pre-LT Treatment**	
Prior Resection (*n* = 161)	15 (0.9%)
Prior TACE (*n* = 148)	16 (1.0%)
Chemotherapy (*n* = 158)	50 (31.6%)
Cisplatin (*n* = 142)	32 (22.5%)
Doxorubicin (*n* = 144)	28 (19.4%)
5-fluorouracil (*n* = 142)	18 (12.7%)
Vincristine (*n* = 144)	18 (12.5%)
Sorafenib (*n* = 144)	10 (6.9%)
Bevacizumab (*n* = 144)	4 (2.8%)
Gemcitabine (*n* = 144)	3 (2.0%)
Oxaliplatin (*n* = 144)	3 (2.0%)
Irinotecan (*n* = 144)	2 (1.3%)
Cyclophosphamide (*n* = 144)	2 (1.0%)
Bleomycin (*n* = 144)	2 (1.3%)
**Post-LT Treatment**	
Chemotherapy (*n* = 126)	30 (23.8%)
Doxorubicin (*n* = 119)	14 (11.7%)
Cisplatin (*n* = 119)	13 (10.9%)
5-fluorouracil (*n* = 117)	7 (5.9%)
Vincristine (*n* = 119)	6 (5.0%)
Sorafenib (*n* = 119)	4 (3.4%)
Cyclophosphamide (*n* = 119)	3 (2.5%)
Bevacizumab (*n* = 119)	3 (2.5%)
Carboplatin (*n* = 125)	3 (2.4%)
Nivolumab (*n* = 119)	2 (1.6%)
Capecitabine (*n* = 119)	2 (1.6%)
Gemcitabine (*n* = 119)	1 (0.8%)
Oxaliplatin (*n* = 119)	1 (0.8%)
Irinotecan (*n* = 119)	1 (0.8%)
Etoposide (*n* = 119)	1 (0.8%)
Immunosuppression (*n* = 67)	
Corticosteroids (*n* = 57)	56 (98.2%)
Tacrolimus (*n* = 62)	44 (71.0%)
Cyclosporine (*n* = 59)	24 (40.7%)
Mycophenolate mofetil (*n* = 62)	12 (19.4%)
Sirolimus (*n* = 59)	3 (5.1%)
Anti-lymphocyte globulin (*n* = 59)	2 (3.4%)
Everolimus (*n* = 59)	1 (1.7%)

PFIC: progressive familial intrahepatic cholestasis, A1AT: a1-antithrypsin, DGUOK: deoxyguanosine kinase, MPV: mitochondrial inner membrane, MRCD: mitochondrial respiratory chain disorder, IFALD: intestinal failure-associated liver disease, CESD: cholesteryl ester storage disease, MDR: multidrug resistance, NCL: neuronal ceroid lipofuscinosis, ADA: adenosine deaminase deficiency.

**Table 3:** Cause of Death After Liver Transplantation for Pediatric Hepatocellular Carcinoma.

Cause of Death	Total (*n* = 60)
Tumor recurrence	22 (36.7%)
Chronic allograft rejection	5 (8.3%)
Sepsis	5 (8.3%)
Primary non-function	3 (5.0%)
Cytomegalovirus infection	2 (3.3%)
Aspiration pneumonia	1 (1.7%)
Respiratory distress and multi-organ failure	1 (1.7%)
Budd-Chiari syndrome	1 (1.7%)
Cardiac arrhythmia	1 (1.7%)
Dialysis-related complication	1 (1.7%)
Hepatic artery thrombosis	1 (1.7%)
Intraoperative cardiac arrest	1 (1.7%)
Metabolic disease	1 (1.7%)
Motor vehicle crash	1 (1.7%)
Post-transplant lymphoproliferative disease	1 (1.7%)
Ruptured pseudoaneurysm	1 (1.7%)
Portal vein thrombosis and intra-operative death during retransplantation	1 (1.7%)
Liver failure (patient also had tumor recurrence)	1 (1.7%)
Unknown	10 (16.7%)

## Data Availability

The data were extracted from already published studies and thus can be found publicly available in the respective full-text articles.

## Supplementary Materials

The following supporting information can be downloaded at: https://www.mdpi.com/article/10.3390/cancers14051294/s1, Table S1. PRISMA (Preferred Reporting Items for Systematic Reviews and Meta-analysis) Checklist.
